# Prognostic values of geriatric nutrition risk index on elderly patients after spinal tuberculosis surgery

**DOI:** 10.3389/fnut.2023.1229427

**Published:** 2023-08-08

**Authors:** Yong Huang, Ruibang Wu, Qinghong Xia, Limin Liu, Ganjun Feng

**Affiliations:** ^1^Department of Orthopedic Surgery and Orthopedic Research Institute, West China Hospital, Sichuan University, Chengdu, Sichuan, China; ^2^Operating Room of Anesthesia Surgery Center, West China Hospital, Sichuan University/West China School of Nursing, Sichuan University, Chengdu, Sichuan, China

**Keywords:** geriatric nutritional risk index, elderly, spinal tuberculosis surgery, clinical outcome, complications

## Abstract

**Background:**

Spinal tuberculosis (STB) is a significant public health concern, especially in elderly patients, due to its chronic and debilitating nature. Nutritional status is a critical factor affecting the prognosis of STB patients. The geriatric nutritional risk index (GNRI) has been established as a reliable predictor of adverse outcomes in various diseases, but its correlation with surgical outcomes in elderly STB patients has not been studied.

**Objective:**

The study aimed to assess the prognostic value of the GNRI in elderly patients with STB who underwent surgery.

**Methods:**

We conducted a retrospective analysis of medical records of elderly patients (65 years or older) diagnosed with active STB who underwent surgical treatment. Data collection included patient demographics, comorbidities, clinical history, laboratory testing, and surgical factors. GNRI was calculated using serum albumin levels and body weight. Postoperative complications were observed and recorded. The patients were followed up for at least 1 year, and their clinical cure status was assessed based on predefined criteria.

**Results:**

A total of 91 patients were included in the study. We found that a GNRI value of <98.63 g/dL was a cutoff value for predicting unfavorable clinical prognosis in elderly STB patients undergoing surgery. Patients with a low GNRI had higher Charlson Comorbidity Index scores, were more likely to receive red blood cell transfusions, and had a higher prevalence of overall complications, particularly pneumonia. The unfavorable clinical prognosis group had lower GNRI scores compared to the favorable prognosis group. Multivariate analysis showed that lower GNRI independently predicted unfavorable clinical outcomes in elderly STB patients.

**Conclusion:**

The study concluded that the GNRI is a valuable biomarker for predicting prognosis in elderly STB patients undergoing surgical intervention. Patients with lower GNRI scores had worse outcomes and a higher incidence of complications.

## Introduction

Spinal tuberculosis (STB), a relatively common form of tuberculosis, primarily affects the spine and has been traced back to ancient times (1). It accounts for 1 to 3% of all tuberculosis cases and is present in approximately 50 to 60% of musculoskeletal tuberculosis cases (2, 3). STB is becoming a significant public health concern with the increase in global migration and drug-resistant strains (4, 5). The disease often has a chronic and occult onset, leading to delayed diagnosis (6). Clinical manifestations vary but commonly include back pain, sometimes accompanied by systemic symptoms (7). The destruction of vertebral bodies and the development of kyphosis can result in severe complications, such as paraplegia, making it one of the most hazardous pathological changes in the musculoskeletal system (8). STB in the elderly has increased due to the aging of the population (9). However, managing elderly STB becomes more complex due to compromised physiology, medical comorbidities, and potential drug interactions (10–12). In addition, nerve damage and paralysis can occur even during the early stage of this disease due to spinal degeneration and stenosis (13). Surgery in the elderly carries additional risks, including longer operative times, increased blood loss, and the need for extended segment fixation due to osteoporosis and degenerative spine (14, 15). Therefore, it is of great significance to find an indicator to evaluate elderly STB patients at admission for predicting potential adverse prognosis.

The anticipation of surgical intervention for STB is affected by many variables, among which nourishing status is the critical factor (16). Two nutritional biomarkers, controlling nutritional status score and prognostic nutrition index, have been proven to be independent predictors of adverse outcomes of STB postoperatively (17). The geriatric nutritional risk index (GNRI) developed by Bouillanne et al. (18) is an assessment tool used to evaluate the nutritional status and risk of malnutrition in elderly individuals. A mounting of literature has proven that GNRI is a good predictive value for adverse outcomes such as morbidity, mortality, length of hospital stay, and complications in different diseases (19–22). However, the correlation between the GNRI and surgical outcomes in elderly STB patients has not been reported. The aim of this study was to evaluate whether the perioperative GNRI can be used as an effective biomarker for predicting prognosis in elderly STB patients undergoing surgical intervention.

## Methods

### Patients

A retrospective analysis was conducted on the medical records of patients diagnosed with active STB who underwent surgical treatment within the period from February 2012 to October 2021. Ethical approval was obtained from our institutional review board (No. 2020–318), ensuring adherence to the Helsinki Declaration and relevant guidelines and regulations. Written informed consent was obtained from all participants in this study. The inclusion criteria were as follows: (1) elderly patients (65 years or older) with confirmed active STB through radiography, histopathology, and bacteriology, (2) patients who underwent primary surgical treatment involving debridement, bone graft fusion, and internal fixation for STB, (3) pre-surgical use of antituberculous drugs for 2–4 weeks, and (4) regular blood testing before and after surgery. In addition, the exclusion criteria were as follows: (1) incomplete laboratory testing, (2) cases where lesion specimens indicated pyogenic infection caused by bacteria other than tuberculosis, and (3) follow-up period of less than 1 year. Initially, a total of 131 patients were identified for potential inclusion in this study. However, after careful assessment, 5 patients who had previously undergone debridement, bone graft fusion, and internal fixation prior to admission, 13 patients with incomplete clinical data, and 22 patients who were lost to follow-up were excluded. Ultimately, a cohort of 91 patients fulfilling all the inclusion criteria was included in the final analysis.

### Data collection

Data pertaining to patient age, sex, Charlson Comorbidity Index (CCI) score, clinical history of hypertension, smoking and drinking, duration of illness and anti-tuberculosis agents before surgery, laboratory testing erythrocyte sedimentation rate (ESR) and C-reactive protein (CRP), and length of hospital day were collected. We also collected two important parameters including serum albumin levels and body weight to calculate GNRI. The formula for calculating GNRI is as follows: GNRI = [1.489 × serum albumin (g/L)] + [41.7 × (current body weight/ideal body weight)] (18). The ideal body weight is calculated based on the Lorentz formula for ideal body weight in elderly individuals, which takes into account the individual’s height and gender (23). The clinical presentation of STB factors including the number of involved vertebrates, location of lesions, abscess, kyphosis deformity, extra-osseous lesions, and neurologic status assessed by the Frankel scoring system were included. Surgical-associated factors including operative time, estimated blood loss, surgical approach, fused vertebrae, and blood transfusion were collected. Postoperative complications were observed and recorded: pneumonia, pneumothorax, wound-associated complications (incision infection and wound dehiscence), venous thrombus embolism (VTE), cerebrospinal fluid(CSF) leakage, urinary tract infection (UTI) and recurrence.

### Preoperative and postoperative management

The patients received a standardized regimen of anti-TB drugs, including isoniazid, rifampicin, ethambutol, and pyrazinamide, which started at least 2 weeks prior to surgery. Concurrent diseases were managed according to routine protocols, and the patients’ hepatic and renal function, electrolyte levels, and cardiopulmonary function were evaluated before the surgery. Surgery was performed when the erythrocyte sedimentation rate (ESR) and C-reactive protein (CRP) levels returned to normal or significantly decreased, and anemia was completely corrected. Following surgery, the same anti-TB drug regimen as before surgery was continued for a minimum of 12 months. Regular follow-up was conducted at monthly intervals during the first 3 months post-surgery, and then at 3–6 month intervals thereafter. The follow-up was conducted through various means, including telephone, outpatient visits, and review of medical records. At the one-year postoperative follow-up, the patients were categorized into either a favorable prognosis group or an unfavorable prognosis group based on clinical cure criteria. Patients who did not meet the clinical cure criteria continued with anti-tuberculosis treatment. X-rays, computed tomography (CT), and magnetic resonance imaging (MRI) scans of the surgical site were reviewed at 6 and 12 months postoperatively to assess bone graft fusion, internal fixation stability, correction of deformity, and the presence of a paravertebral abscess.

### Clinical cure standard

(1) The clinical manifestations of STB were absent for a period exceeding 3 months, (2) Various levels of improvement in neurological dysfunction were observed, (3) There were no indications of infection in the affected area of the spine, and the sinus had healed without any discharge, (4) The ESR and CRP levels were within the normal range on three consecutive occasions, and (5) Imaging tests showed the absence of abscesses, necrotic bone tissue, or fusion with bone grafts.

### Statistics

For the analysis of continuous variables, an independent t-test was employed, while categorical comparisons were conducted using chi-square tests. To identify cutoff values for the GNRI and assess its diagnostic accuracy, a receiver operating characteristic (ROC) curve was constructed, and the Youden index and area under the curve (AUC) were utilized. All significant risk factors were included in a multivariable logistic regression model to determine independent predictors of STB, and odds ratios (ORs) with 95% confidence intervals (CIs) were calculated. Statistical analyzes were performed using IBM Corp.’s SPSS statistical software version 20. A *p*-value less than 0.05 was considered statistically significant.

## Results

**Figure 1:**
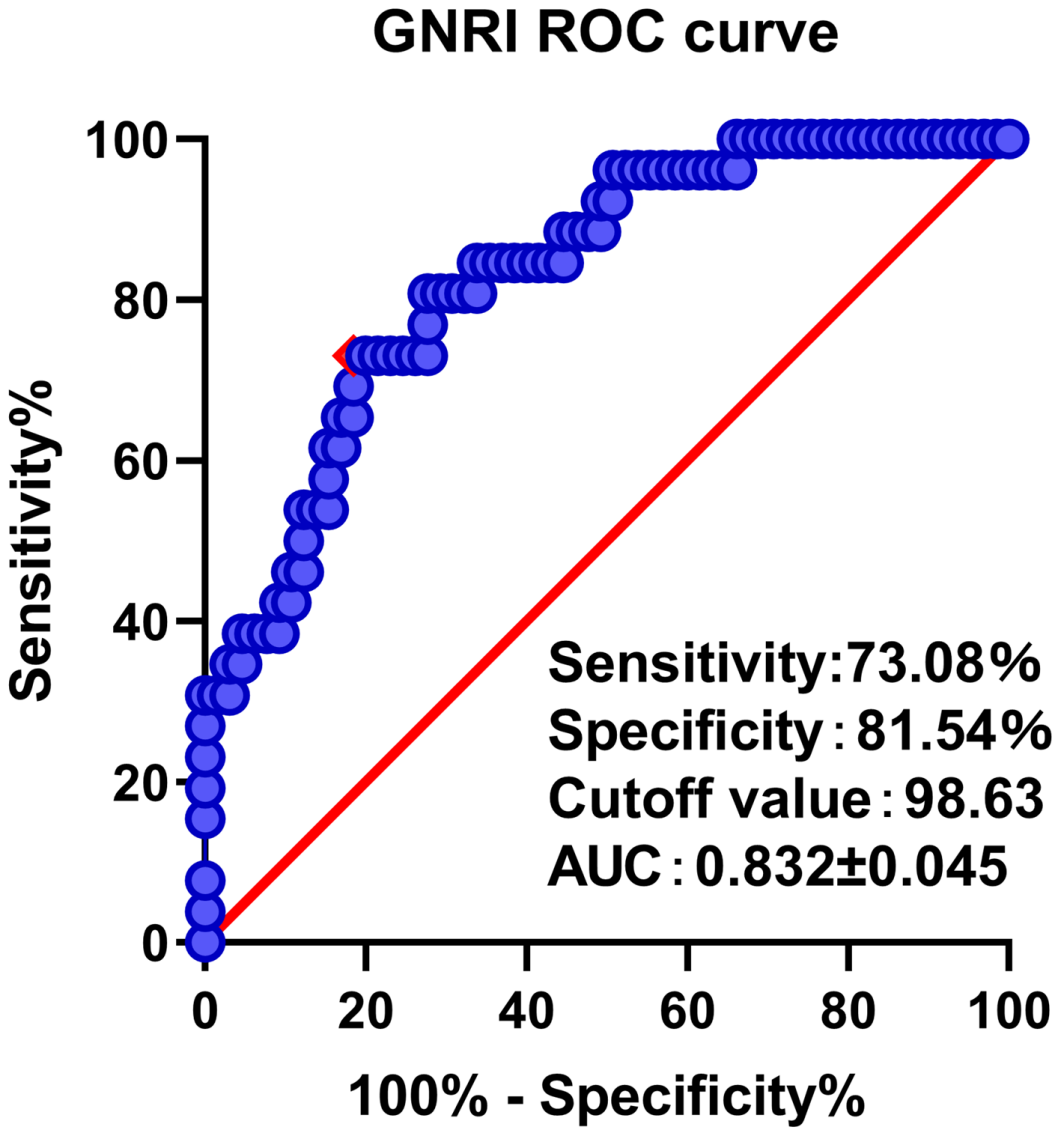
ROC curve analysis and AUC of the GNRI. The red point indicates the cutoff points determined by the Youden index.

A total of 91 patients (Mean age 70.49 ± 3.91 years, 44 males, 47 females) were included in the study (Table 1). ROC curve analysis demonstrated GNRI <98.63 g/dL as a cut-off value for predicting unfavorable clinical prognosis with a sensitivity of 73.08% and a specificity of 81.54% (Figure 1). The AUC of the GNRI score was 0.832 (95% CI 0.745–0.919). Thus, for the final analysis, 31 patients were included in the low GNRI cohort, and 60 patients were included in the high GNRI cohort (Table 1). The low GNRI group had a higher CCI score (2.06 ± 0.73 vs. 1.40 ± 1.14, *p* < 0.001), more likely to receive red blood cell transfusions treatment (48.39% vs. 25.00%, *p* = 0.024) and a higher prevalence of overall complications (74.19% vs. 28.13%, p < 0.001). Among the complications, pneumonia (29.03% vs. 8.33%, *p* = 0.022) was the most common and statistically different.

**Table 1:** Characteristic in patients with different GNRI.

Characteristic	Total (*n* = 91)	GNRI		*p*-value
		Low-GNRI (<98.6), *n* = 31	High-GNRI (≥98.6), *n* = 60	
Demographic				
Age (years)	70.49 ± 3.91	70.94 ± 3.98	70.27 ± 3.88	0.442
Sex (male, %)	44 (48.35)	14 (45.16)	30 (50.00)	0.662
CCI	1.21 ± 1.06	2.06 ± 0.73	1.40 ± 1.14	0.004*
Hypertension, *n* (%)	28 (30.77)	10 (32.26)	18 (30.00)	0.825
Smoking history, *n* (%)	10 (10.99)	2 (6.45)	8 (13.33)	0.521
Drinking history, *n* (%)	13 (14.29)	4 (12.90)	9 (15.00)	1.000
Duration of illness (months)	4.13 ± 2.26	3.90 ± 2.10	4.25 ± 2.35	0.491
Duration of anti-TB agents (months)	1.27 ± 0.75	1.24 ± 0.72	1.30 ± 0.78	0.729
ESR (mm/1 h)	48.71 ± 13.67	48.85 ± 16.89	48.64 ± 11.84	0.946
CRP (mg/L)	28.55 ± 6.03	29.80 ± 6.91	27.90 ± 5.46	0.156
Length of hospital day	11.05 ± 3.66	11.64 ± 4.08	10.75 ± 3.41	0.271
Vertebral body involvement				
Abscess, *n* (%)	54 (59.34)	18 (58.06)	36 (60.00)	0.859
Kyphotic deformity, *n* (%)	32 (35.16)	10 (32.26)	22 (36.67)	0.676
Number of involed verterbrae, *n* (%)				0.533
1	7 (7.69)	2 (6.45)	5 (8.33)	
2–3	69 (75.82)	22 (70.98)	47 (78.33)	
>3	15 (16.48)	7 (22.58)	8 (13.33)	
Location of lesions, *n* (%)				0.911
Cervical	11 (12.09)	4 (12.90)	7 (11.67)	
Thoracic	41 (45.05)	13 (41.94)	28 (46.67)	
Lumbosacral	39 (42.86)	14 (45.16)	25 (41.67)	
Franked scale, *n* (%)				0.183
B	2 (2.20)	0 (0.00)	2 (3.33)	
C	12 (13.19)	3 (9.68)	9 (15.00)	
D	25 (27.47)	6 (19.35)	19 (31.67)	
E	52 (57.14)	22 (70.97)	30 (50.00)	
Extra-osseous lesions, *n* (%)	18 (19.78)	6 (19.35)	12 (20.00)	0.074
Surgical factors				
Operative time (minutes)	196.80 ± 77.66	202.08 ± 93.45	194.07 ± 75.01	0.644
Estimated blood loss (mL)	627.25 ± 253.19	650.44 ± 277.09	615.27 ± 241.49	0.533
Surgical approach, *n* (%)				0.432
Anterior	32 (35.16)	11 (35.48)	21 (35.00)	
Posterior	47 (45.05)	14 (45.16)	33 (55.00)	
Combined	12 (13.19)	6 (19.35)	6 (10.00)	
Fused vertebrae	4.53 ± 1.39	4.52 ± 1.48	4.53 ± 1.36	0.956
Blood transfusion, *n* (%)				
RBC	30 (32.97)	15 (48.39)	15 (25.00)	0.024*
Plasma	17 (18.68)	9 (29.03)	8 (13.33)	0.069
Complications, *n* (%)	40 (43.96)	23 (74.19)	17 (28.33)	0.000*
Pneumonia	14 (15.38)	9 (29.03)	5 (8.33)	0.022*
Pneumothorax	6 (6.59)	4 (12.90)	2 (3.33)	0.194
CSF leakage	2 (2.20)	1 (3.00)	1 (1.67)	0.554
VTE	5 (5.49)	3 (9.68)	2 (3.33)	0.439
UTI	7 (7.69)	3 (9.68)	4 (6.67)	0.924
Wound associated complications	5 (5.49)	3 (9.68)	2 (3.33)	0.439
Recurrence	1 (1.10)	0 (0.00)	1 (1.67)	1.000

*p < 0.05, the difference between the Low-GRNI group and the High-GRNI group was significant.

Depending on the clinical cure standard at 1 year, twenty-six (28.57%) patients had an unfavorable result, and sixty-five (71.43%) patients had a favorable result in our research. The GNRI in the unfavorable clinical prognosis group was significantly lower compared with the favorable clinical prognosis group (96.21 ± 6.95 vs. 106.69 ± 8.19, *p* < 0.001) (Table 2). The correlation between clinical prognosis and basic clinical characteristics was explored by a univariate analysis 1 year postoperatively. The results showed that patients in the unfavorable group were older (71.92 ± 4.30 vs. 69.92 ± 3.62, *p* = 0.027), had a higher CCI score (2.03 ± 0.87 vs. 1.46 ± 1.19, *p* = 0.018), were more inclined to red blood cell transfusions therapy (53.85% vs. 24.61%, *p* = 0.007) and had a higher rate of pneumonia (34.62% vs. 7.69%, *p* = 0.024) and pneumothorax (19.23% vs. 1.54%, *p* = 0.009) complications (Table 2). In the multivariate analysis of unfavorable outcomes, lower GNRI (odds ratio [OR] 1.141, 95% confidence interval [CI] 1.050–1.241, *p* = 0.002) was the only variable that significantly predicted unfavorable STB clinical prognosis outcomes (Table 3).

**Table 2:** Univariate analysis of included patients by one-year clinical prognosis in general.

Factors	Unfavorable clinical prognosis (*n* = 26)	Favorable clinical prognosis (*n* = 65)	*p*-value
Demographic			
Age (years)	71.92 ± 4.30	69.92 ± 3.62	0.027*
Sex (male, %)	12 (45.15)	32 (49.23)	0.791
CCI	2.03 ± 0.87	1.46 ± 1.19	0.018*
Hypertension, *n* (%)	7 (26.92)	21 (32.31)	0.615
Smoking history, *n* (%)	2 (7.69)	8 (12.31)	0.791
Drinking history, *n* (%)	6 (23.08)	7 (10.77)	0.236
Duration of illness (months)	3.73 ± 1.87	4.29 ± 2.40	0.287
Duration of anti-TB agents (months)	1.12 ± 0.66	1.33 ± 0.79	0.245
ESR (mm/1 h)	52.11 ± 12.57	47.35 ± 13.96	0.135
CRP (mg/L)	29.57 ± 6.28	28.14 ± 5.92	0.308
Length of hospital day	11.69 ± 3.82	10.80 ± 3.58	0.295
GNRI	96.21 ± 6.95	106.69 ± 8.19	0.000*
Vertebral body involvement			
Abscess, *n* (%)	15 (57.69)	39(60.00)	0.840
Kyphotic deformity, *n* (%)	10 (38.46)	22 (33.85)	0.677
Number of involed verterbrae, *n* (%)			0.574
1	2 (7.69)	5 (7.69)	
2–3	18 (69.23)	51 (78.46)	
>3	6 (23.08)	9 (13.85)	
Location of lesions, *n* (%)			0.414
Cervical	5 (19.23)	6 (9.23)	
Thoracic	11 (42.31)	30 (46.15)	
Lumbosacral	10 (38.46)	29 (44.62)	
Franked scale, *n* (%)			0.671
B	0 (0.00)	2 (3.08)	
C	3 (11.54)	9 (13.85)	
D	7 (26.92)	18(27.69)	
E	16 (61.54)	36 (55.38)	
Extra-osseous lesions, *n* (%)	6 (23.08)	12 (18.46)	0.618
Surgical factors			
Operative time (minutes)	197.70 ± 82.48	196.44 ± 76.31	0.945
Estimated blood loss (mL)	632.03 ± 304.81	625.33 ± 232.04	0.910
Surgical approach, *n* (%)			0.542
Anterior	9 (34.62)	23 (35.38)	
Posterior	12 (46.15)	35 (53.85)	
Combined	5 (19.23)	7 (10.78)	
Fused vertebrae	4.42 ± 1.45	4.57 ± 1.38	0.654
Blood transfusion, *n* (%)			
RBC	14 (53.85)	16 (24.61)	0.007*
Plasma	7(26.92)	10 (15.38)	0.202
Complications, *n* (%)	25 (96.15)	15 (23.08)	0.000*
Pneumonia	9 (34.62)	5 (7.69)	0.024*
Pneumothorax	5 (19.23)	1 (1.54)	0.009*
CSF leakage	1 (3.85)	1 (1.54)	0.492
VTE	3 (11.54)	2 (3.08)	0.275
UTI	4 (15.38)	3 (4.62)	0.191
Wound associated complications	3 (11.54)	2 (3.08)	0.275
Recurrence	0 (0.00)	1 (1.54)	1.000

*p < 0.05, the difference between the unfavorable clinical prognosis group and the favorable clinical prognosis was significant.

**Table 3:** Results of multivariable analysis association with unfavorable clinical prognosis.

Factors	*β*	OR	95%CI	*p* value
Age(years)	−0.133	0.875	0.752–1.108	0.085
CCI	−0.342	0.710	0.388–1.301	0.268
GNRI	0.132	1.141	1.050–1.241	0.002*
RBC blood transfusion	0.783	2.195	0.628–7.670	0.218
pneumonia	1.184	3.267	0.683–15.622	0.138
Pneumothorax	3.264	26.155	0.539–1268.275	0.099

*p < 0.05, the factor is statistically significant.

## Discussion

As the understanding and techniques for treating STB continue to advance, the primary goal of surgical treatment has shifted towards eradicating TB lesions, relieving spinal nerve compression, reconstructing spinal stability, addressing spinal deformities, and ultimately improving the quality of life for elderly patients (24, 25). However, malnutrition and frailty, which are prevalent and interconnected among geriatric individuals, pose potential risk factors for adverse postoperative outcomes (14, 26). In the context of spine surgery, malnutrition has been associated with complications such as delirium, surgical site infections, and wound dehiscence (27, 28). Among elderly patients with cervical spondylotic myelopathy, preoperative malnutrition has been closely linked to the increased occurrence of cervical kyphosis following laminoplasty (29). Additionally, individuals with latent tuberculosis are prone to tuberculosis reactivation and progression to active tuberculosis when malnutrition sets in and cellular immune function declines (30). Active tuberculosis requires increased energy expenditure, necessitating an additional 20–30% of daily caloric intake (31). Consequently, malnutrition is considered a widespread risk factor for tuberculosis. Recurrent infections further exacerbate nitrogen loss and worsen nutritional status, rendering individuals more susceptible to infections. This vicious cycle poses particular harm to geriatric patients (32). Therefore, our study focuses on evaluating the prognostic value of preoperative nutritional status in elderly patients undergoing STB surgery. This retrospective, single-center study revealed that geriatric patients with lower GNRI scores had worse outcomes and experienced a higher incidence of complications compared to those with higher GNRI scores at the 1-year follow-up. Multivariate analysis demonstrated that GNRI scores independently predicted adverse postoperative outcomes in elderly patients with STB.

The GNRI, an index used to predict mortality risk based on nutritional status, is applicable to all patients, including elderly individuals with age-related malnutrition (18). Extensive literature supports the strong correlation between the GNRI and various nutritional evaluations, enabling it to effectively forecast short- and long-term clinical outcomes (33, 34). This index relies on objective parameters such as serum albumin concentration and weight loss. In addition, the GNRI stands out as a cost-effective and efficient tool for assessing nutritional status upon admission, making it suitable for routine evaluation in population-based settings (35). Moreover, due to its association with nutrition-related factors, the GNRI can aid in the prevention of complications related to underlying malnutrition, such as bedsores and infections, ultimately improving the prognosis for undernourished patients (36). Numerous studies have demonstrated the utility of the GNRI for screening malnutrition in patients with chronic diseases like peritoneal dialysis, heart failure, cancer, and stroke. In these cases, a low GNRI is indicative of an unfavorable prognosis, including poor functional outcomes, higher prevalence of complications, and increased mortality rates (34, 37–39). Building on our study, the GNRI can effectively guide perioperative treatment and nursing for elderly patients with STB, facilitating personalized care. Comprehensive assessment and management of nutritional status before surgery are crucial in preventing postoperative functional decline and unfavorable surgical outcomes, as malnutrition can be modified as a risk factor prior to the procedure. Additionally, patients with gastrointestinal dysfunction should receive early provision of parenteral nutrition to ensure targeted caloric support.

It is important to acknowledge the limitations of our study. Firstly, it is a retrospective, single-center study with a small sample size. Secondly, while trace elements have been implicated in influencing tuberculosis infection and immunity, our study did not include their measurement. Due to the low content of vitamins and trace elements, sensitive technology is necessary for accurate analysis, and precautions must be taken to avoid contamination during measurement. Moreover, our assessment only focused on preoperative nutritional status, neglecting postoperative nutritional evaluation. Consequently, further prospective studies are required to explore the correlation between the GNRI and the prognosis of elderly patients with STB.

## Conclusion

This study demonstrated that a lower GNRI is linked to negative outcomes in elderly patients with STB. The GNRI serves as a cost-effective and easily accessible set of biomarkers that can assist in identifying individuals with poor prognoses, who would benefit from early nutritional intervention. These significant findings should be further investigated through prospective studies to validate their implications.

## Data availability statement

The raw data supporting the conclusions of this article will be made available by the authors, without undue reservation.

## Ethics statement

The studies involving humans were approved by Ethics Committee of West China Hospital of Sichuan University. The studies were conducted in accordance with the local legislation and institutional requirements. The participants provided their written informed consent to participate in this study.

## Author contributions

YH and RW conceptualized and designed the study and drafted the initial manuscript. QX carried out the initial analyzes, and reviewed and revised the manuscript. GF and LL coordinated and supervised data collection, critically reviewed, and revised the manuscript for important intellectual content. All authors contributed to the article and approved the submitted version.

## Funding

The authors acknowledge the funding support from the National Natural Science Foundation of China (Nos. 82072434 and 82272546). The organizations had no involvement in the study design, collection, analysis, or interpretation of data, in the writing of the manuscript, or in the decision to submit the manuscript for publication.

## Conflict of interest

The authors declare that the research was conducted in the absence of any commercial or financial relationships that could be construed as a potential conflict of interest.

## Publisher’s note

All claims expressed in this article are solely those of the authors and do not necessarily represent those of their affiliated organizations, or those of the publisher, the editors and the reviewers. Any product that may be evaluated in this article, or claim that may be made by its manufacturer, is not guaranteed or endorsed by the publisher.

## Abbreviations

GNRI: geriatric nutrition risk index
STB: spinal tuberculosis surgery
MRI: magnetic resonance imaging
CT: computed tomography
BMI: body mass index
CCI: Charlson Comorbidity Index
CSF: cerebrospinal fluid
VTE: venous thrombus embolism
UTI: urinary tract infection
ROC: receiver operating characteristic
AUC: area under the curve
ORs: odds ratios
CIs: confidence intervals
